# Idarucizumab in dabigatran-treated patients with acute stroke: a review and clinical update

**DOI:** 10.3389/fneur.2024.1389283

**Published:** 2024-05-16

**Authors:** Senta Frol, Janja Pretnar Oblak, Mišo Šabovič, George Ntaios, Pawel Kermer

**Affiliations:** ^1^Department of Vascular Neurology, University Medical Center Ljubljana, Ljubljana, Slovenia; ^2^Faculty of Medicine, University of Ljubljana, Ljubljana, Slovenia; ^3^Department of Vascular Disorders, University Medical Center Ljubljana, Ljubljana, Slovenia; ^4^Faculty of Medicine, Department of Internal Medicine, School of Health Sciences, University of Thessaly, Larissa, Greece; ^5^Department of Neurology, Nordwest-Krankenhaus Sanderbusch, Friesland Kliniken GmbH, Sande, Germany; ^6^University Medical Center Göttingen, Göttingen, Germany

**Keywords:** idarucizumab, clinical trials, real-world data, clinical update, clinical practice

## Abstract

Idarucizumab is an antibody fragment specific for the immediate reversal of dabigatran anticoagulation effects. The use of idarucizumab is approved for dabigatran-treated patients suffering from life-threatening or uncontrolled bleeding and those in need of urgent surgery or invasive procedures. Data from randomized controlled clinical trials and real-world experience provide reassuring evidence about the efficacy and safety of idarucizmab use in patients with acute stroke. In this narrative review, we summarize the available real-world evidence and discuss the relevance and importance of idarucizumab treatment in acute stroke patients in everyday clinical practice. In addition, we also discuss special issues like prothrombin complex concentrate application as an alternative to idarucizumab, its application before endovascular therapy, sensitivity of thrombi to lysis, and necessary laboratory examinations.

## Introduction

Dabigatran—one of the four direct oral anticoagulants (DOACs) approved in most countries of the world—is a thrombin inhibitor that was tested in standard and reduced doses in the Randomized Evaluation of Long-Term Anticoagulation Therapy (RE-LY) (1) trial. This trial showed superior safety for both doses, superior efficacy for a standard dose, and at least comparable efficacy for reduced dose when compared to warfarin (1). These results were confirmed in multiple real-world studies (2–4). Dabigatran in standard dose was also tested in the Efficacy and Safety of Dabigatran Compared to Warfarin for 6 Month Treatment of Acute Symptomatic Venous Thromboembolism (RE-COVER) study, showing similar effects on acute venous thromboembolism (VTE) recurrence and a lower risk of bleeding compared with warfarin for the treatment of acute VTE (5).

Acute ischemic stroke (AIS) in dabigatran-treated patients is an infrequent event with yearly incidences of 0.9% for the standard dose (6, 7) and 1.3% for the reduced dose (1). For intracranial hemorrhage (ICH), annual incidences are even lower with 0.10% for the standard dose and 0.12% for the reduced dose, respectively (1). If AIS does occur, intravenous thrombolysis (IVT) is highly effective if given promptly. The benefits of IVT are proportionally higher when administered as early as possible. For patients suffering from ICH, prevention of hematoma growth, efficient blood pressure control, normalizing the level of anticoagulant activity, and timely neurosurgical procedures, if indicated, are relevant for clinical outcomes.

All DOACs are approved for primary and secondary stroke prevention in patients with non-valvular atrial fibrillation (AF) and also for VTE (8). In AF patients, the necessity for anticoagulation is lifelong if the CHADS2-VASc score is ≥2 in male patients and ≥ 3 in female patients.

Hence, a reversal agent is advantageous when patients require emergency interventions or need an urgent procedure. In patients suffering acute stroke, a reversal agent with high efficacy, lacking safety concerns, that improves clinical outcomes and facilitates the regain of eligibility for urgent interventions is very valuable (9).

Idarucizumab is a specific agent for dabigatran with immediate and complete reversal of its anticoagulant effects within minutes and is ready to use as a bolus injection in a standard dose of 5 g intravenously (10, 11). No intrinsic procoagulant or anticoagulant effects have been reported to date. In healthy male human volunteers, idarucizumab is safe and well tolerated in the absence of dabigatran, with no effect on coagulation parameters (12). The efficacy and safety of idarucizumab in the treatment of acute major bleeding was evaluated in the REVERSal Effects of idarucizimab on Active Dabigatran (RE-VERSE AD) study (11). It is indicated in dabigatran-treated patients suffering from life-threatening or uncontrolled bleeding and those in need of urgent surgery or invasive procedures (11, 13). The current guidelines of the European Stroke Organization (ESO) (14), the European Hearth Rhythm Association (EHRA) (15), and the American Heart Association/American Stroke Association (AHA/ASA) (16) recommend the use of idarucizumab in dabigatran-treated patients suffering acute ICH. In dabigatran-treated patients with the last dose taken ≤48 h before AIS symptoms, the 2021 EHRA (15) and the 2021 ESO (17) guidelines recommend IVT after dabigatran reversal with idarucizumab in patients who are eligible for IVT and have no other contraindications. Current guideline recommendations are not based on data from randomized controlled trials (RCTs) (9).

There is a growing body of published real-world cases of idarucizumab use in dabigatran-treated patients with acute stroke providing data on efficacy and safety of its treatment. In our systematic reviews published in 2021 (18, 19), we analyzed real-world data on the efficacy and safety of idarucizumab use before IVT in dabigatran-treated patients with AIS (18) and the safety of its use in ICH (19). Rates of hemorrhagic transformation (HT), symptomatic intracranial hemorrhage (SICH), mortality, and National Institutes of Health Stroke Scale (NIHSS) reduction were comparable with previously published studies in non-anticoagulated patients (20, 21), regardless of age and stroke severity upon admission (18). Furthermore, we found that the NIHSS score at admission appeared to be an independent predictor of mortality (18). The data were updated by the summary of new real-world data of idarucizumab use in AIS until 22/07/2022, additionally confirming the safety and efficacy of this treatment strategy (22). In this context, there is accumulating evidence that idarucizumab use before IVT is safe (23). Therefore, this therapeutic strategy should be used when indicated.

In a recently published international, multicentre, retrospective cohort study conducted by Meinel et al. (24), authors evaluated the risk of SICH associated with the use of IVT in DOAC-treated patients, including patients treated with dabigatran who were receiving idarucizumab before IVT. In their study, 33,207 patients with AIS who were receiving IVT were included: 832 patients receiving DOAC treatment and 32,375 patients without DOAC treatment. The primary outcome of the study was SICH, and the secondary outcomes were any radiological ICH and functional independence (defined as a modified Rankin Scale (mRS) score of ≤2 at 90 days). The authors reported that the unadjusted rate of SICH was 2.5% in patients receiving DOACs compared with 4.1% in patients without DOAC treatment. After adjustment, the authors concluded that the recent use of DOACs was not associated with an increased risk of SICH development, regardless of idarucizumab reversal in dabigatran-treated patients (24). It is also worth noting the data on the safe and effective use of idarucizumab before tenecteplase—a fibrinolytic not approved in Europe so far—for the use in AIS patients (25). Data published in 2021 (19) and 2023 (22) showed an in-hospital mortality of 9.7–11.4% in dabigatran-treated patients suffering from ICH and receiving idarucizumab (19), which is lower compared to known data on mortality in patients treated with oral anticoagulants suffering from ICH without the use of reversal agents (26).

In New Zealand, where dabigatran has a very high prescription rate (25), all patients treated with stroke reperfusion therapies are entered into a mandatory online national registry. In an article published by New Zealand clinicians, Barber et al. (25) reported that the use of idarucizumab prior IVT in dabigatran patients (*n* = 51) was safe with similar clinical outcomes to routinely managed AIS patients without DOAC treatment (*n* = 1,285), despite a 22-min door-to-needle time delay, and concluded that their study provides Class III evidence of IVT benefit in this patient cohort, which is otherwise excluded from this treatment option (25). Regarding shorter door-to-needle times, data from prehospital idarucizumab use in mobile stroke units (27, 28) also provided encouraging safety and efficacy results.

Despite these advantages, after almost a decade of its use, dabigatran, in the current times, has a rather low prescription rate. In older adults with geriatric profiles, who represent the majority of DOAC users, the size of dabigatran capsules might be challenging, especially in the presence of mild dysphagia. Moreover, administration via enteral feeding tubes is not recommended due to large variations in drug exposure (29), and pharmacokinetics and pharmacodynamics in the elderly often differ substantially from the younger population. In line with this, dabigatran has the largest interindividual variability compared to other DOACs (30)—a finding that might be reflected by the fact that patient comorbidities such as kidney function, muscle mass, and comedication with P-glycoprotein (P-gp) inhibitors instead of age (>75) are not applied as dose adjustment criteria. In real life, this uncertainty regarding dose adjustment often leads to off-label low dosages. A recent study confirmed that, among all DOACs, dabigatran dose was most frequently prescribed as sn off-label drug (15.3%), and it was also the most frequently underdosed one (9.6%) (31). This result is even more concerning since exposure outside of on-therapy ranges is known to be related to stroke and bleeding events. Nevertheless, real-life clinical data support the safety of dabigatran even in high-risk groups (32, 33), which is further increased by the availability of its specific antidote idarucizumab.

In this commentary review, we summarize and discuss the relevance and importance of idarucizumab treatment for acute stroke patients in everyday clinical practice, spanning almost a decade of its use.

## Clinical trials on idarucizumab use in acute stroke patients

The efficacy and safety of idarucizumab in the treatment of acute major bleeding were evaluated in the RE-VERSE AD study (11). A total of 98 patients with different ICH types were included, which revealed an immediate and complete reversal of dabigatran anticoagulation effects after idarucizumab application. Complete reversal of diluted thrombin time and ecarin clotting time was observed in 100 and > 90% of evaluable patients, respectively; thrombin time reversed to normal levels post-idarucizumab, and no patients required two doses of idarucizumab. The 30-day mortality rate with idarucizumab was 16.4% (11), which is much lower than without idarucizumab in the RE-LY RCT (1). There are no data on IVT in AIS patients treated with idarucizumab from RCTs.

## Real-world data on idarucizumab use in acute stroke patients

RE-VECTO program was a global surveillance project in which the pattern of idarucizumab use was explored, indicating that off-label idarucizumab use was very rare (26).

In our previous systematic reviews (18, 19), we investigated the safety and efficacy of idarucizumab treatment in dabigatran-treated patients with AIS (18) and the safety of idarucizumab treatment in dabigatran-treated patients with ICH (19).

Updated literature search for additional case reports and case series, trials, reviews, and guidelines extending the period of our previous exploration to 21/12/2023 using the same strategy and inclusion criteria, data extraction and outcomes, methodology, and statistical analysis (22) retrieved 16 articles (8, 24, 34–47) [six reviews (34–39), two original research articles (24, 40), one postmarketing surveillance study (41), one clinical practice guidelines (8), one case report (42), and five case series (43–47)].

### Idarucizumab use in dabigatran-treated patients with AIS

We updated the literature search for additional case reports and case series, trials, reviews, and guidelines, extending the period of our previous exploration (22) to 21/12/2023 using the following terms: “idarucizumab” and “stroke” or “ischemic stroke.” In addition, we searched the references of related letters and editorials to identify other potentially eligible studies. To be eligible for the present analysis, the studies had to be full-text articles published in the English language. Duplicates were excluded.

Six articles with 326 patients suffering AIS while on dabigatran and treated with idarucizumab followed by IVT (24, 42, 43, 45–47) were identified. Two studies provided individual patient data from 7 patients (42, 46), whereas 4 case series reported outcomes from 319 patients (24, 43, 45, 47). None of the patients described by Amin et al. (42) suffered HT, suffered SICH, or died. The mean NIHSS at admission was 10 and 6.5 at discharge (42). Similarly, in a study by Dai et al. (46), the authors reported an in-hospital mortality rate of 0. For the case series, in-hospital mortality was reported in three papers (43, 45, 47) and was 11.94% on average, while it was not reported in a study written by Meinel et al. (24). HT was documented in 28 out of 310 patients (9.0%) (24, 43, 47), while the number of patients suffering from HT was not documented in a study done by Kikule et al. (45). SICH developed in 10 out of 319 patients (3.1%) (24, 43, 45, 47). The median NIHSS reduction between admission and discharge was 6 points in the case series reported by Włodarczyk et al. (47) and 5 points in the case series reported by Kikule et al. (45), while NIHSS at admission and discharge was not reported in another two studies (24, 43).

One review article with meta-analysis addressed the use of idarucizumab in dabigatran-treated patients with AIS before IVT (38), in which 407 out of 3,610 (11%) patients received idarucizumab before IVT administration. Ghannam et al. reported that there was no significant difference in the SICH rate based on idarucizumab administration compared to those who had not received it (38).

### Idarucizumab use in dabigatran-treated patients with ICH

We updated the literature search for additional case reports and case series, trials, reviews, and guidelines extending the period of our previous exploration (22) to 21/12/2023 using the terms “idarucizumab” and “stroke” or “hemorrhagic stroke” or “intracranial bleeding.” In addition, we searched the references of related letters and editorials to identify other potentially eligible studies. To be eligible for the present analysis, the studies had to be full-text articles published in the English language. Duplicates were excluded.

Two articles with 23 patients suffering from ICH while on dabigatran and treated with idarucizumab (44, 46) were identified. A total of one study provided individual patient data from 8 patients (46), whereas 1 case series reported outcomes from 15 patients (44). None of the patients reported in a study by Dai et al. (46) died in the hospital; a total of 2 out of 8 patients (20%) suffered thromboembolic complications (46). In the case series reported by Kuklik et al. (44), 4 patients out of 15 patients died in the hospital (26.7%), and none suffered from thromboembolic complications.

Five review articles (34–37, 39) addressed the use of idarucizumab regarding its use in ICH. A meta-analysis done by Chaudhary et al. (36) revealed an anticoagulation reversal rate of 82%, a mortality rate of 11%, and a thromboembolic rate of 5% after idarucizumab application in patients with ICH (36), which is in line with the RE-VERSE AD study results. Based on the analysis of available published data, all authors support the use of idarucizumab in ICH (34–37, 39), which is also reflected in clinical practical guidelines written by Joglar et al. (8). In a review by Al Aseri et al. (34), authors additionally addressed the concern of limited availability of specific reversal agents in various countries and stressed the urgent need of availability and accessibility for developing local guidelines to direct their use (34). The final analysis from the Japanese postmarketing surveillance study in which 804 patients were included (41) confirmed previously reported data from an interim analysis (48) revealing the efficacy and safety of idarucizumab treatment in dabigatran-treated patients suffering from ICH (41).

In an original article written by Spyropoulos et al. (40), the authors evaluated the cost-effectiveness of idarucizimab versus andexanet-alfa (AA) use in ICH patients. The authors found out that patients treated with idarucizmab appeared to incur a lower total cost compared with patients treated with AA (40). Their explanation for the lower idarucizumab total cost was the lower cost of idarucizumab itself; furthermore, they claimed that patients who were treated with AA had higher severity of illness and higher proportion of ICH, which resulted in more intensive treatment approaches (40).

## Specific issues

### Idarucizumab versus prothrombin complex concentrate (PCC) in ICH

For patients on dabigatran therapy presenting with ICH, the initial treatment strategy should focus on blood pressure lowering and the prevention of hematoma growth by antagonizing dabigatran activity. Guidelines (14–16, 49, 50) and consensus statement (51) for the treatment of ICH in patients taking dabigatran are quite homogeneous and recommend treatment with idarucizumab as first-line therapy. If a specific reversal agent is not available, guidelines recommend the administration of a PCC or an activated PCC for patients treated with DOACs who develop life-threatening bleeding (14, 52). However, it is crucial to remember that all PCC components act upstream of thrombin inhibition by dabigatran (53, 54). Hence, PCC effects remain unspecific and competitive. Moreover, *ex vivo* experiments with different DOACs indicated that PCC could not reestablish thrombin formation (1).

Idarucizumab has no known effects on the coagulation system aside from inactivating dabigatran. Regarding the RE-VERSE AD study (11) and real-world data (18, 22), the rate of thrombotic events after idarucizumab application is low. Thromboembolic complications after PCC use in DOAC-treated patients with ICH are of great concern and are discussed intensively in current literature (55). According to our knowledge, no head-to-head studies are comparing the efficacy and safety of PCC versus idarucizumab use in dabigatran-treated patients suffering from ICH.

Lack of safety issues and the clinical benefits when applying idarucizumab should outweigh concerns regarding medical expenses. Therefore, PCC should only be used in case idarucizumab is not available. Restricted availability and concerns regarding cost-effectiveness still limit the clinical use of idarucizumab in low- and middle-income countries.

Tranexamic acid could be a treatment option in areas where neither idarucizumab nor PCC might be available, but the current ESO guidelines recommend against using tranexamic acid outside of trials (14).

### Endovascular treatment (EVT) in AIS patients on dabigatran therapy with large vessel occlusion (LVO)

EVT is the therapy of choice in AIS patients with LVO (15). RCTs on acute invasive revascularization therapy in DOAC-treated patients are lacking.

The additional value of IVT before EVT in patients with LVO has been extensively debated over the last decade, leading to reassuring results of non-omitting IVT before EVT, unless there are contraindications against IVT (56, 57). According to accumulating evidence that idarucizumab use before IVT may be effective and safe, this therapeutic strategy should also be used in patients with LVO before EVT, especially if a patient is initially referred to a center not performing EVT (drip and ship concept).

### Sensitivity of thrombi treated with dabigatran prior to idarucizumab and to IVT

Regarding high success rates of IVT after a reversal in dabigatran-treated patients found in different cohorts (13, 58) as well as in our updated report, it is reasonable to assume higher sensitivity of thrombi in patients treated with anticoagulation before lysis, as suggested by Pretnar et al. (59). In the clinical setting, the lysability of stroke thrombi strongly depends on thrombus structure (60). The thrombus structure in stroke is typically heterogeneous and contains fibrin, platelets, red blood cells, von Willebrand factor, and neutrophil extracellular traps. In fibrin- and platelets-rich parts of the thrombi, a dense, compacted network of thin fibers with entrapped platelets is present, whereas, in red blood cell-rich parts, there is only a loose, poorly compacted network of thick fibrin fibers with red blood cells (61). Fibrin- and platelet-rich parts are much less susceptible to lysis with tissue plasminogen activator (t-PA) than the red blood cell-rich parts of thrombi (61). Histological studies suggest that cardioembolic thrombi are predominantly composed of fibrin- and platelet-rich parts, while noncardioembolic thrombi are mainly associated with red blood cell-rich parts (62). Therefore, cardioembolic thrombi may be less susceptible to thrombolysis with t-PA.

Thrombin plays an important role in the modulation of thrombus structure (63). At high thrombin concentrations, a dense network of thin fibers forms, which enables the formation of fibrin-rich thrombi with entrapped platelets. In contrast, at low thrombin concentrations, a loose, porous fibrin network with thick fibrin fibers forms, which facilitates the formation of red blood cell-rich thrombi (64). Overall, it appears that dabigatran may be able to affect the structure of stroke thrombi and thus susceptibility to thrombolysis by lowering thrombin concentration. All thrombin inhibitors probably have an important influence on thrombi structure (65). This issue and its clinical relevance need to be further investigated.

Under laboratory conditions, plasma clots that form in the presence of dabigatran have been shown to have an altered structure that is more susceptible to thrombolysis, with a looser, less rigid, and more permeable fibrin network with thicker fibers (64). In addition, dabigatran reduces activated thrombin-activatable fibrinolysis inhibitor (TAFI) activity. Both mechanisms, TAFI-dependent and nondependent, could increase the susceptibility of thrombi that form in the presence of dabigatran to lysis with t-PA (64).

Idarucizumab has been shown to have no effect on t-PA-induced thrombolysis in human plasma *in vitro* (12). In addition, idarucizumab has no procoagulant or anticoagulant effect; therefore, it cannot enhance coagulation (12, 66).

## Laboratory exams

In our previous article (22), we proposed a standard operating procedure (SOP) regarding idarucizumab use in dabigatran-treated patients with AIS and ICH, also including data regarding special laboratory exams. If dabigatran was taken more than 48 h before the hospital admission in patients with AIS with normal renal function and/or normal hemostaseological parameters, IVT can be given without dabigatran reversal by idarucizumab. If taken within 48 h or laboratory exams like thrombin time (TT), diluted thrombin time (dTT), and hemoclot test or dabigatran plasma levels or renal function are pathologic, as well as if those laboratory values are not available, we recommend idarucizumab application in case of AIS or ICH (22). However, some authors deem it mandatory to have baseline hemoclot levels and point-of-care (POC) tests before idarucizumab treatment (24). Based on our updated literature search and additional encouraging safety and efficacy results of prehospital idarucizumab application in mobile stroke units (27, 28), extending the time window to idarucizumab treatment and subsequent IVT is not justified.

## Practical guidelines: standard operating procedure (SOP)

There is accumulating evidence suggesting that dabigatran reversal with idarucizumab in the setting of AIS and ICH may be safe and effective. To assist clinicians in managing dabigatran-treated patients suffering from AIS and ICH, we have already proposed an SOP (22). Decision-making on the use of idarucizumab should be based on three relevant pieces of information: the last time of dabigatran intake, special laboratory exams, and renal function (22). If dabigatran intake was less than 48 h before AIS symptoms onset, if laboratory exams are abnormal, if renal function is impaired, or if there is uncertain value/time of last dabigatran intake and if the patient is an eligible candidate lacking other contraindications for IVT, patients should be treated with IVT after idarucizumab reversal. In the case of ICH, we suggest idarucizumab application if dabigatran intake was less than 48 h before the onset of symptom, if laboratory exams are abnormal, if renal function is impaired, or if there is uncertain value/time of last dabigatran intake.

## Conclusion

**Figure 1:**
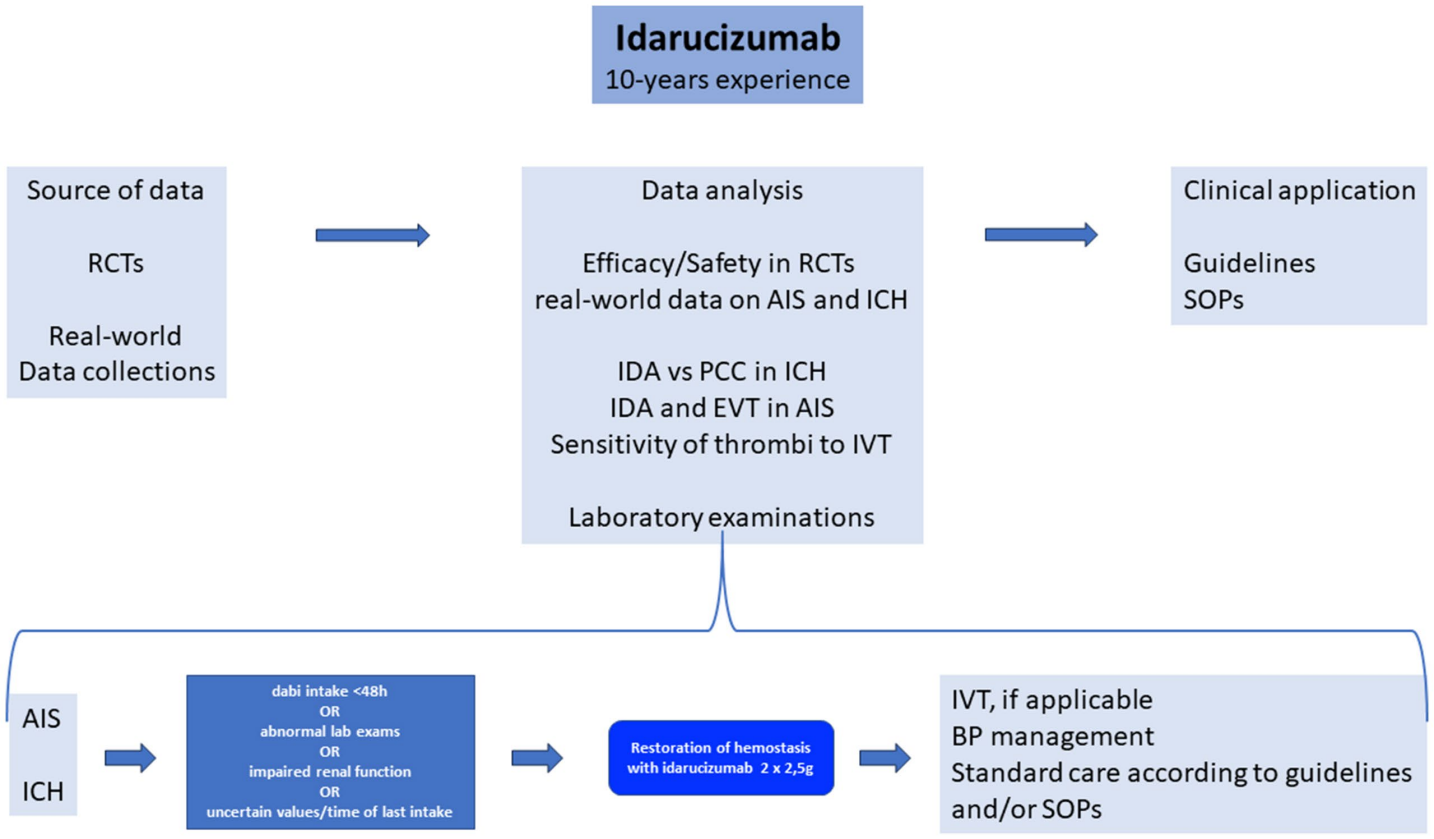
Idarucizumab 10-year experience. RCTs, randomized clinical trials; AIS, acute ischemic stroke; ICH, intracranial hemorrhage; dabi, dabigatran; lab, laboratory; IDA, idarucizumab; PCC, prothrombin complex concentrate; EVT, endovascular treatment; IVT, intravenous thrombolysis; BP, blood pressure; SOPs, standard operating procedures; dabi, dabigatran.

This review of all real-world evidence summarizes the current literature regarding idarucizumab treatment in acute stroke patients over almost a decade of its use. The source of data were RCTs and real-world data collections to analyze the efficacy and safety of idarucizmab use in RCTs and provide real-world data on its use in AIS and ICH (Figure 1). As specific issues, we highlight the use of idarucizumab versus PCC in ICH, idarucizumab and EVT in AIS, and the sensitivity of thrombi to IVT (Figure 1). Furthermore, we discuss the laboratory examinations that are needed before idarucizumab treatment. We propose specific SOPs and guidelines (Figure 1). In the case of AIS or ICH, if dabigatran intake is <48 h, if laboratory exams are abnormal, renal function is impaired, or the time of last dabigatran intake is unknown or uncertain, restoration of hemostasis with idarucizumab 2 × 2.5 g is suggested, followed by IVT, if applicable in AIS, intense blood pressure lowering in ICH, and standard care of AIS and ICH according to guidelines and/or SOPs (Figure 1).

## Author contributions

SF: Writing – original draft, Writing – review & editing. JP: Writing – review & editing. MŠ: Writing – review & editing. GN: Writing – review & editing. PK: Writing – original draft, Writing – review & editing.
